# Pre-Existing Medical Conditions: A Systematic Literature Review of a Silent Contributor to Adult Drowning

**DOI:** 10.3390/ijerph19148863

**Published:** 2022-07-21

**Authors:** Amy E. Peden, Danielle H. Taylor, Richard C. Franklin

**Affiliations:** 1 School of Population Health, Faculty of Medicine and Health, University of New South Wales, Kensington, NSW 2052, Australia; a.peden@unsw.edu.au; 2 College of Public Health, Medical and Veterinary Sciences, James Cook University, Townsville, QLD 4811, Australia; danielle.taylor@my.jcu.edu.au

**Keywords:** pre-existing medical condition, drown, epilepsy, cardiac, injury, suicide, dementia, depression, ischaemic heart disease, seizure

## Abstract

Medical conditions can increase drowning risk. No prior study has systematically reviewed the published evidence globally regarding medical conditions and drowning risk for adults. MEDLINE (Ovid), PubMed, EMBASE, Scopus, PsycINFO (ProQuest) and SPORTDiscus databases were searched for original research published between 1 January 2005 and 31 October 2021 that reported adult (≥15 years) fatal or non-fatal drowning of all intents and pre-existing medical conditions. Conditions were grouped into the relevant International Classifications of Diseases (ICD) codes. Eighty-three studies were included (85.5% high-income countries; 38.6% East Asia and Pacific region; 75.9% evidence level III-3). Diseases of the nervous system (n = 32 studies; 38.6%), mental and behavioural conditions (n = 31; 37.3%) and diseases of the circulatory system (n = 25; 30.1%) were the most common categories of conditions. Epilepsy was found to increase the relative risk of drowning by 3.8 to 82 times, with suggested preventive approaches regarding supervised bathing or showering. Drowning is a common suicide method for those with schizophrenia, psychotic disorders and dementia. Review findings indicate people with pre-existing medical conditions drown, yet relatively few studies have documented the risk. There is a need for further population-level research to more accurately quantify drowning risk for pre-existing medical conditions in adults, as well as implementing and evaluating population-level attributable risk and prevention strategies.

## 1. Introduction

Drowning has been described as an underexplored threat to public health [1]. Drowning, the process of experiencing respiratory impairment due to immersion or submersion in liquid, has both fatal and non-fatal outcomes, with or without morbidity [2]. Drowning may also be unintentional, intentional or of undetermined intent. Unintentional drowning alone was estimated to claim the lives of 295,000 people around the world in 2017, with the true estimate likely to be significantly higher with the inclusion of transportation and disaster-related drowning [3]. Though less is known about drowning due to intentional self-harm [4,5], many countries also report high drowning rates due to suicide [6,7,8].

Chronic medical conditions are becoming more common [9]. This phenomenon effects low and high-income countries alike. Research on unintentional drowning risk among children has identified epilepsy [10,11] and autism spectrum disorder as conditions posing an increased risk of drowning [12,13,14]. Several studies have explored the role of chronic disease on drowning risk among older people—an age group of growing concern with respect to drowning due to an aging population [15,16]. Studies among the elderly population have highlighted the risk for drowning of conditions such as dementia, sarcopenia, epilepsy, cardiac conditions and depression [5,11,17,18,19,20]. However, little is known regarding the drowning risk associated with pre-existing medical conditions and the interventions recommended to reduce this risk.

To date, no study has systematically reviewed the literature to examine the role of pre-existing medical conditions on adult drowning risk. This review will address the following research questions concerning the link between pre-existing medical conditions, drowning and drowning risk:

1. Which pre-existing medical conditions have been reported in adult (aged 15 years and older) drowning cases?
2. Which pre-existing medical conditions does the literature suggest impact the risk of drowning?
3. In which population (s) do pre-existing medical conditions increase drowning risk and burden?
4. What drowning reduction strategies are recommended in the literature?

## 2. Materials and Methods

A systematic review of peer-reviewed literature was undertaken to identify and critically analyse studies reporting drowning and chronic medical conditions in adults (aged 15 years and older) and was prospectively registered with PROSPERO (#CRD42020190605). The study followed the Preferred Reporting Items for Systematic Reviews and Meta-Analysis (PRISMA) guidelines [21]. From the search results, the PRISMA statement was used to identify, screen and determine eligibility for the included studies.

Peer-reviewed literature published in English between 1 January 2005 and 31 October 2021 was identified from searches of MEDLINE (OVID), PubMed, EMBASE, Scopus, PscyINFO (ProQuest) and SPORTDiscus databases. Search terms were intentionally broad, and no qualification of methodology or publication type was applied in the first search to capture all relevant article sets. Search terms included “drown*”, “adult”, “medical”, “disease” and various medical conditions. Where possible, terms and medical conditions were mapped to MESH terms. The Boolean search strings utilised for this study are described in Appendix A.

Literature was limited to a publication date of 2005, as this was the year the current drowning definition was established [2]. Studies of human drowning and chronic medical conditions were included regardless of outcome (fatal or non-fatal) and intent (unintentional, intentional self-harm, undetermined intent). Non-fatal drowning was defined in line with the Non-Fatal Drowning Categorisation Framework (NDCF) [22]. The full inclusion and exclusion criteria for the study are displayed in Table 1. Studies were included where data could be extracted for pre-existing medical conditions for people aged 15 years and older. Medical conditions were included if there was a history of the condition for the person who drowned, even if the condition was not indicated at autopsy. Conditions noted at autopsy but unknown at the time of the drowning incident were also included. Studies were excluded if they only reported acute conditions (e.g., a broken bone during the drowning incident). Case reports were included if they contained data for six or more drowning cases, regardless of the presence of a pre-existing medical condition. (Table 1).

Using Covidence literature screening software, the independent dual screening of title and abstract was undertaken, with conflicts resolved via consensus between the two reviewers. The process was repeated for the full-text review. Data were extracted using a custom-built Microsoft Excel spreadsheet. Data extracted included age group and number of participants, number of drowning incidents including by intent and outcome, study type, name of medical condition(s), number, proportion and/or rate of those who drowned with medical condition and statistical measure of risk (i.e., Chi-square tests of independence, relative risk, odds ratio). Medical conditions were identified by extracting key medical findings (i.e., medical condition, pathophysiology findings) presented in the literature. Specific medical conditions were coded to the relevant category within the International Classification of Diseases (ICD) 10 category [23]. The categories and examples of conditions coded to each category taken from included studies are shown in Table 2. For the top three most common groups of conditions, the specific conditions within the groupings were further categorised as depicted in Table 2 [24,25]. Where studies did not report on a specific medical condition, these were coded to a grouping called “all pre-existing medical conditions”.

Risk factors were defined if statistical tests identified a significant link between the medical condition and risk of drowning or drowning outcome (i.e., Chi-square tests of significance, odds ratio, relative risk). Prevention strategies were extracted as free text if proposed, implemented and/or evaluated specific to drowning. Prevention strategies were coded as primary, secondary or tertiary prevention [26] and against the corresponding level within the Hierarchy of Control [27]. Quality of evidence was also assessed using the National Health and Medical Research Council (Australia) Levels of Evidence [28]. Levels of evidence range from Level I (a systematic review of Level II studies (randomised controlled trial)) to Level IV (case studies with either post-test or pre-test/post-test outcomes). Region and income levels of countries represented in included studies were assessed using the World Bank open data country profiles [29].

## 3. Results

**Figure 1:**
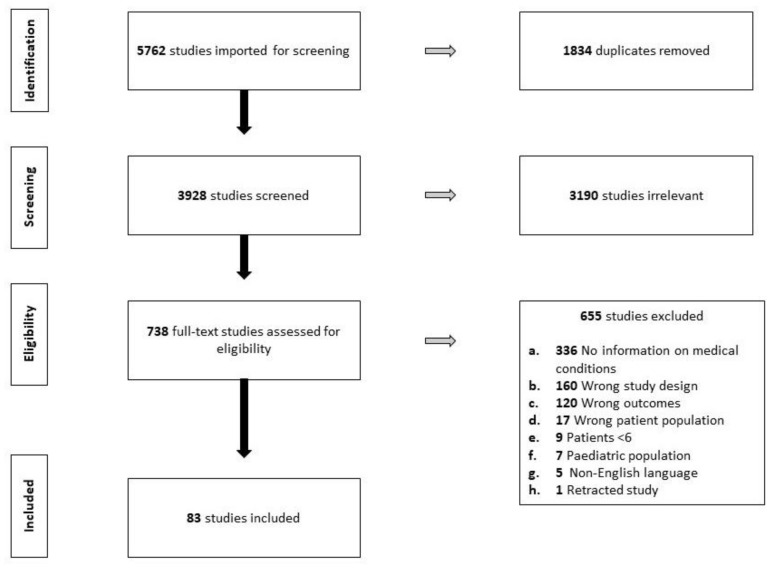
PRISMA flow chart.

Initial searches identified 5762 studies. After the removal of 1834 duplicates, a total of 3928 studies were screened at the title and abstract stage. After the removal of studies not meeting the inclusion criteria, 738 full-text studies were assessed for eligibility. Following a full text review, 83 studies were included for data extraction (Figure 1).

Included studies predominately reported data from high-income countries (n = 71; 85.5%). The largest numbers of included studies were from the World Bank region groupings East Asia and the Pacific (n = 32; 38.6%) and Europe and Central Asia (n = 28; 33.7%). The majority of the included studies were assessed at a level of evidence of III-3 (n = 63; 75.9%). There were 48 studies (57.8%) that reported unintentional drowning, and 79 (95.2%) reporting fatal drowning. The study characteristics of the full list of included studies can be found in Appendix B.

With respect to grouped medical conditions, diseases of the nervous system [7,11,19,20,30,31,32,33,34,35,36,37,38,39,40,41,42,43,44,45,46,47,48,49,50,51,52,53,54,55,56,57] and mental and behavioural conditions [7,37,42,43,54,56,57,58,59,60,61,62,63,64,65,66,67,68,69,70,71,72,73,74,75,76,77,78,79,80,81,82] were the most commonly reported categories of medical conditions in drowning, identified in 32 studies (38.6%) and 31 studies (37.3%), respectively. This was followed by diseases of the circulatory system (n = 25 studies; 30.1% of all included studies) [19,43,46,49,53,54,55,57,63,76,83,84,85,86,87,88,89,90,91,92,93,94,95,96,97,98] (Table 3).

There were 13 studies that reported all pre-existing medical conditions [7,43,49,53,63,69,98,103,104,105,106,107,108]. The proportion of drowning involving pre-existing medical conditions ranged from 2.8% with chronic illness among fatal land motor vehicle drownings in Finland [106] to 24.6% of elderly (defined as 65 years and over) drowning patients in South Korea (fatal and non-fatal) reporting chronic illness (such as diabetes, hypertension and hepatitis) [69]. In the South Korean study, a significantly higher (*p* < 0.001) of elderly patients had chronic disease (24.6%) compared with the rest of the adult population who drowned (3.3%)[69]. A total population study of unintentional drowning fatalities in Canada identified that 67.3% of all adults 65+ years reported one or more accompanying chronic conditions [98]. Pre-exiting medical conditions were also prevalent in a study of intentional drowning death in Australia, found in 83.1% of deaths [7].

Seizure disorders (including epilepsy) were the most commonly reported condition within the diseases of the nervous system category, reported in 23 studies [11,19,20,30,32,33,34,35,37,38,39,40,43,44,45,46,47,49,52,53,54,55,56]. Epilepsy was found to occur in 11% of sudden deaths in hot bathtubs in Japan [20] and 9.6% of adult unintentional fatal drownings in Bangladesh [37]. Among those with epilepsy, drowning accounted for 83.3% of accidental injury deaths in Bangladesh [44] yet just 0.05% of seizure-related fatal unintentional injuries in Thailand [38] and 0.4% of hospitalised epilepsy deaths in the USA [39].

Almost half (49.1%) of all people in Portugal and the United Kingdom (UK) surveyed with Parkinson’s Disease reported having experienced a non-fatal drowning [48]. Drowning deaths of people with dementia who die after going missing or wandering span from 11.3% to 42.1% [36,51] (Table 4).

Within the mental and behavioural conditions category, psychotic disorders (n = 15 studies) and mood disorders (n = 13 studies) were the two most commonly reported types of conditions implicated in cases of drowning. Drowning accounted for 9% of suicidal deaths in patients with schizophrenia in Taiwan [74]. Among those with psychotic disorders, drowning deaths varied from a high of 20.9% among people with personality disorders in Sweden [60] to a low of 1.3% of intentional drowning deaths in Australia [7]. Forty percent of psychiatric patients who died by suicide in South Korea drowned with psychotic disorders [75]. Psychotic disorders were present in 27.2% of patients who died from intentional drowning within one year of contact with mental health services in the UK [65].

Mood disorders (including bipolar and depression) were present in 61.3% of drowning deaths (both intentional and unintentional) in the Madurai region of India [79] and in 45.0% of intentional fatal drowning among psychiatric patients who suicided in South Korea [75]. A further eight studies reported substance abuse disorders. It should be noted that substance use disorders were present in 75.3% of suicidal drowning deaths in Australia [64] and 15.6% of drowning deaths in France [76] (Table 5).

Heart arrythmias (or related conditions) were the most commonly reported condition within the diseases of the circulatory system category, reported in eight studies [19,63,83,84,86,91,95,96]. Heart arrythmias were present in 22.9% of “unexplained” drowning deaths referred for a cardiac channel molecular autopsy in the USA [91] and 22.2% of diving-related drowning fatalities in Australia [95]. Among older people, heart arrythmias were present in 21.7% of bathtub drownings among people aged 65+ years in Canada [63] and 15.6% of the same cohort in Australia [19].

Ischaemic heart disease was identified in five included studies [53,55,85,89,90]. Two studies were from Greece, finding that ischaemic heart disease was present in 87.9% [85] and 51.8% of drowning deaths, respectively [89]. Two other studies reporting bath-related deaths found that ischaemic heart disease was present in 34.2% of bath-related deaths in Japan [53] and 73.7% in South Korea [55].

Among other circulatory system conditions, hypertensive heart disease was present in 66.7% of drowning deaths among those competing in triathlons in the USA [83,84], and atherosclerosis was found in 20% of those who drowned with a pre-existing medical condition in Greece [85] (Table 6).

**Table 5:** Studies reporting conditions within the mental and behavioural conditions category.

Condition	Reference	Country	Study Population	Age Group	Intent	Outcome	% Who Drowned	% Who Drowned with Med Conditions	% Who Drowned with Condition
Anxiety disorder	Ahlm et al., 2015 [59]	Sweden	All drowning deaths in Sweden	16–85 years	I	F	0.8%	-	-
	Cenderadewi et al., 2019 [7]	Australia	Intentional drowning deaths	All ages	I	F	2.4%	2.9%	-
	Fang et al., 2015 [61]	China	Individuals with psychiatric disorder who committed suicide by drowning	10–89 years	I	F	-	1.9%	-
Behavioural disorder	Bjorkenstam et al., 2016 [60]	Sweden	Total population with personality disorders Sweden	15–64 years	I	F	-	1.1%	-
	Ljusic et al., 2018 [70]	Serbia	Deaths among those with mental disorders, somatic disorders or no registered disorder	-	I	F	14.1%	100%	
Cognitive function	Bjorkenstam et al., 2016 [60]	Sweden	Total population with personality disorders Sweden	15–64 years	I	F	-	2.2%	-
	Fang et al., 2015 [61]	China	Individuals with psychiatric disorder who committed suicide by drowning	10–89 years	I	F	-	9.7%	-
	Kim et al., 2021 [42]	South Korea	Deaths of people with a disability	All ages	U	F	-	3.0%	-
Mood disorder	Aaltonen et al., 2019 [58]	Finland	All suicide after first lifetime psychiatric hospitalisation for depression	18+ years	I	F	-	7.4%	-
	Ahlm et al., 2015 [59]	Sweden	All drowning deaths in Sweden	16–85 years	I	F	9.5%	-	-
	Bjorkenstam et al., 2016 [60]	Sweden	Total population with personality disorders Sweden	15–64 years	I	F	-	31.9%	-
	Cenderadewi et al., 2019 [7]	Australia	Intentional drowning deaths	All ages	I	F	20.2%	24.3%	-
	Fang et al., 2015 [61]	China	Individuals with psychiatric disorder who committed suicide by drowning	10–89 years	I	F	-	64.5%	-
	Hunt et al., 2006 [65]	UK	Suicide with recent (within 1 year) contact with mental health services	0–75+ years	I	F	49.0%	-	-
	Lee et al., 2019 [69]	South Korea	Fatal drowning	18+ years	I,U, Und	F	18.7%	-	-
	Maity et al., 2020 [71]	India	Drowning deaths	0–70 years	Und	F	4.9%	-	-
	Nishida et al., 2015 [73]	Japan	Patients diagnosed with early post stroke depression who died	65–94 years	I	F	70.8%	-	-
	Park et al., 2013 [75]	South Korea	Psychiatric patients who suicide	10+ years	I	F	45.0%	-	-
	Runeson et al., 2010 [77]	Sweden	Completed suicides among those treated for attempted suicide	10+ years	I	F	29.1%	-	-
	Schaffer et al., 2014 [78]	Canada	Suicide in bipolar disorder	All ages	I	F	-	-	2.9%
	Selveraj et al., 2020 [79]	India	Drowning in Madurai Region	All ages	I,U	F	61.3%	-	-
Personality disorder	Bjorkenstam et al., 2016 [60]	Sweden	Total population with personality disorders Sweden	15–64 years	I	F	-	100.0%	-
	Hunt et al., 2006 [65]	UK	Suicide with recent (within 1 year) contact with mental health services	All ages	I	F	3.7%	-	-
Psychosexual disorder	Fang et al., 2015 [61]	China	Individuals with psychiatric disorder who committed suicide by drowning	10–89 years	I	F	-	1.0%	-
Psychotic disorder	Ahlm et al., 2015 [59]	Sweden	All drowning deaths in Sweden	16–85 years	I	F	4.2%	-	-
	Bjorkenstam et al., 2016 [60]	Sweden	Total population with personality disorders Sweden	15–64 years	I	F	-	20.9%	-
	Cenderadewi et al., 2019 [7]	Australia	Intentional drowning deaths	All ages	I	F	1.1%	1.3%	-
	Fang et al., 2015 [61]	China	Individuals with psychiatric disorder who committed suicide by drowning	10–89 years	I	F	-	20.2%	-
	Flaig et al., 2013 [62]	Germany	Non-natural death cases autopsied	18–96 years	I	F	9.0%	-	-
	Haines et al., 2010 [64]	Australia	Completed suicides	10–43 years	I	F	80.6%	-	-
	Hunt et al., 2006 [65]	UK	Suicide with recent (within 1 year) contact with mental health services	All ages	I	F	27.2%	-	-
	Kumar et al., 2018 [72]	India	Attempted suicides in psychiatric consultation	10–50 years	I	NF	-	5%	-
	Lee et al., 2019 [69]	South Korea	Fatal drowning	18+ years	I,U, Und	F	7.1%	-	-
	Markarian et al., 2020 [43]	France	Selected patients admitted to ICU for a drowning-related incident	40–74 years	I	F	16.3%	16.6%	-
	Pan et al., 2021 [74]	Taiwan	Suicide mortality in patients with schizophrenia	All ages	I	F	-	-	9.0%
	Park et al., 2013 [75]	South Korea	Psychiatric patients who suicide	10+ years	I	F	40.0%	-	-
	Runeson et al., 2010 [77]	Sweden	Completed suicides among those treated for attempted suicide	10+ years	I	F	11.3%	-	-
	Stemberga et al., 2010 [80]	Croatia	Suicidal drowning deaths	23–86 years	I	F	2.2%	-	-
	Stephenson et al., 2020 [81]	Australia	Drowning deaths in urban section of the River Torrens	18–76 years	I,U	F	32.4%	-	-
	Tellier et al., 2019 [54]	France	Drowning victims along Gironde surf beaches	All ages	U	F	0.9%	10.2%	-
Substance abuse disorder	Ahlm et al., 2015 [59]	Sweden	All drowning deaths in Sweden	16–85 years	I	F	1.4%	-	-
	Bjorkenstam et al., 2016 [60]	Sweden	Total population with personality disorders Sweden	15–64 years	I	F	-	13.2%	-
	Cenderadewi et al., 2019 [7]	Australia	Intentional drowning deaths	All ages	I	F	24.9%	29.9%	-
	Cenderadewi et al., 2019 [7]	Australia	Intentional drowning deaths	All ages	I	F	11.1%	13.4%	-
	Guay et al., 2019 [63]	Canada	Bathtub drownings people aged 65+	65+ years	U	F	3.3%	-	-
	Haines et al., 2020 [64]	Australia	Completed suicides	10–43 years	I	F	75.3%	-	-
	Hunt et al., 2006 [65]	UK	Suicide with recent (within 1 year) contact with mental health services	All ages	I	F	11.7%	-	-
	Reizine et al., 2021 [76]	France	Death after non-fatal drowning in fresh and sea water	All ages	I,U	F	15.6%	21.0%	-
	Williams et al., 2018 [82]	USA	Unintentional drowning episodes, resulting in death or injury among actively serving US armed forces	All ages	U	F,NF	7.0%	-	-
Other disorders	Ahlm et al., 2015 [59]	Sweden	All drowning deaths in Sweden	16–85 years	I	F	1.7%	-	-
	Park et al., 2013 [75]	South Korea	Psychiatric patients who suicide	10+ years	I	F	15.0%	-	-
	Runeson et al., 2010 [77]	Sweden	Completed suicides among those treated for attempted suicide	10+ years	I	F	14.7%	-	-
All mental and behavioural disorders	Ahlm et al., 2015 [59]	Sweden	All drowning deaths in Sweden	16–85 years	I	F	17.6%	-	-
	Cenderadewi et al., 2019 [7]	Australia	Intentional drowning deaths	All ages	I	F	83.1%	-	--
	Fang et al., 2015 [61]	China	Individuals with psychiatric disorder who committed suicide by drowning	10–89 years	I	F	-	1.0%	-
	Fang et al., 2015 [61]	China	Individuals with psychiatric disorder who committed suicide by drowning	10–89 years	I	F	-	2.0%	-
	Guay et al., 2019 [63]	Canada	Bathtub drownings people aged 65+	65+ years	U	F	9.8%	-	-
	Hossain e al, 2017 [37]	Bangladesh	Adult drowning	18+ years	U	F	9.9%	-	-
	Kielty et al., 2015 [66]	Ireland	Individuals who died by probable suicide	18+ years	I	F	23.1%	-	-
	Kim et al., 2021 [42]	South Korea	Deaths of people with a disability	All ages	U	F	-	7.0 *	-
	Koo et al., 2021 [67]	Australia	Data from the Queensland Suicide Register	65+ years	I	F	44.6%	-	-
	Lawes et al., 2021 [68]	Australia	Suicidal deaths along the Australian coast	18+ years	I	F	59.8%	-	-
	Reizine et al., 2021 [76]	France	Death after non-fatal drowning in fresh and sea water	All ages	I,U	F	26.7%	36.0%	-
	Stemberga et al., 2010 [80]	Croatia	Suicidal drowning deaths	23–86 years	I	F	13.4%	-	-

Abbreviations: F = Fatal; I = Intentional; NF = Non-Fatal; OHCA = Out of Hospital Cardiac Arrest; U = Unintentional; UK = United Kingdom; Und = Undetermined; USA = United States of America; * represents crude mortality rate not proportion.

There were 10 risk factors identified from the literature. These included increasing age, being at home, living near water, freshwater, medical conditions, medication (not on correct dose), sex (depending on medical condition), time of day and inpatient vs outpatient treatment (Table 7).

For epilepsy, there is an increase in the risk of drowning from between 3.8 times in the USA [39] to 82 times in China [47]. Specific to epilepsy and drowning, those with epilepsy in a study from the USA were found to be more likely to drown at home than in hospital or at a health care facility [31]; in rural China, those with epilepsy were found to have greater drowning risk if they resided in waterside areas than those living in the mountains [35], to have had epilepsy for a shorter period than those who survived [47] and to have a lower dosage of phenobarbital recorded at time of last follow up than those who survived [47].

By sex, females with personality disorders [60] and schizophrenia [74] were found to be at increased risk of suicidal drowning when compared to males; however, males were found to be at increased risk of dying from drowning with epilepsy [47]. Older age was found to be a risk factor for drowning with pre-existing medical conditions in studies of disability in South Korea (those aged 80+ years) [42], among coastal drowning fatalities in Australia [105] and for elderly patients with diabetes, hypertension and hepatitis in South Korea [69] (Table 7).

There were a total of 17 studies that discussed 26 unique strategies for preventing drowning related to pre-existing medical conditions [7,11,19,31,34,41,44,47,48,53,57,59,68,77,89,91,96]. The majority of strategies were administrative in nature when aligned to the Hierarchy of Control (n = 24; 92.3%) and all were proposed, as opposed to implemented and/or evaluated. Strategies were commonly educational in nature (n = 12 recommendations; 48.0% of all recommendations), followed by testing (n = 6; 24.0%), treatment (n = 3; 12.0%) and policy (n = 3; 12.0%) (Table 8).

**Table 7:** Risk factors related to pre-existing medical conditions and drowning.

Risk Factor	Medical Condition	Note	Reference
Age	Disability Precipitating medical factors Chronic disease	Age related risk for drowning increased as people age, for example for precipitating medical factors younger (15–34 year) males were 3.7 times less likely to drown *.	[42] [105] [69]
Location—home	Epilepsy	People with epilepsy/seizures were more likely to drown at home (RR = 2.35, 95% CI = 1.9–3.0, *p* < 0.001) than people without epilepsy/seizures.	[31]
Location—waterside areas	Epilepsy	Living near water increased the risk compared to those living in the mountains (Hazard Ratio 3.9, 95% CI 1.7–9.2, *p* = 0.002).	[35]
Location—Freshwater	Mental and behavioural condition	When comparing baseline characteristics of the patients according to the salinity of the water, freshwater drowning patients were younger and suffered more often from psychiatric comorbidities (47.9 vs. 19.1%; *p* < 0.001).	[76]
Medical condition	Cardiac disease Cardiomegaly Cardiomyopathy * Chronic conditions Circulatory system Dementia Epilepsy Mental and behavioural condition Mental and Psychotic disorder Schizophrenia	There were 10 medical conditions or groups of conditions that were identified as increasing the risk of drowning. Cardiac disease was found in 14% of all accidental drownings but in none (0%) in the suicide group *p* < 0.05. Cardiomegaly (*p* < 0.05) was higher among those who drowned compared to other causes of sudden or violent death. Drowning cases had significantly lower odds of presenting with cardiomyopathy (*p* < 0.001) than other causes of sudden or violent death. Those with chronic diseases had an OR of 15.1 compared with those who drowned without pre-existing disease. Significantly higher association of CT genotype/allele in drowned people (0.545) than controls (0.279) *p* = 0.008. Drowning was OR 1.55 (95%CI: 0.90–2.69) times more likely among those with dementia than healthy individuals as a suicide method. People with epilepsy drown at a rate between 6.7–82 times greater than the general population, depending on location and age group. Patients with alcoholism more likely to die from drowning. For example, service members with any history of alcohol-related disorder were nearly twice that of those without any history of alcohol-related disorder. Compared with the general population, people with psychotic disorders were 3.28 times (95%CI: 1.16–9.26) more likely to suicide by drowning. Compared to poisoning, psychotic disorder males with a Hazard Ratio 6.2 (95%CI: 3.3 to 11.6) and females with a Hazard Ratio 9.7 (95%CI: 5.3 to 17.8) were more likely to successfully suicide by drowning. Patients diagnosed as having schizophrenia were more likely to commit suicide through drowning than the general population (odds ratio (OR) = 1.48, 95% CI = 1.27–1.73, *p* < 0.001).	[97] [85] [85] [103] [93] [50] [11] [30] [33] [35] [39] [44] [47] [47] [76] [82] [75] [77] [74]
Medication	Epilepsy	The dosage of phenobarbital recorded at the time of last follow-up was lower (*p* < 0.001) in the group who drowned than in those who survived.	[47]
Sex—female	Personality disorders Schizophrenia	Women diagnosed with a PD had the highest SMR for drowning. Compared with schizophrenic men, schizophrenic women were more likely to suicide through drowning (23.8 cases in every 100,000 people; *p* < 0.001).	[60] [74]
Sex—male	Epilepsy	Males with epilepsy were more likely to drown than females with epilepsy (*p* = 0.017).	[47]
Time	Psychotic disorders	Patients were more likely to use suicide methods other than hanging (e.g., OR = 6.7 for jumping, 5.3 for drowning and 2.7 for self-poisoning) between midnight and dawn.	[75]
Treatment	Psychotic disorders	Compared with outpatients, patients who had received inpatient treatment were more likely to use drowning (OR = 3.46; 95%CI: 1.30–9.22; *p* = 0.013) than hanging.	[75]

Abbreviations: COD= cause of death; CI = confidence interval; OR = odds ratio; PD = personality disorder; RR = Relative Risk; SMR = standardised mortality rate. * denotes lower drowning risk.

## 4. Discussion

As the global population ages, the prevalence of comorbidities grows [109]. This systematic literature review shows that drowning occurs in people with pre-existing medical conditions, and that people with pre-existing medical conditions appear to be over-represented in drowning statistics. It also identified several conditions where drowning risk is heightened. Epilepsy was found to increase the relative risk of drowning by between 3.8 [39] and 82 times [47]. Risk factors for drowning in epilepsy included being of male sex [47], drowning at home [31], lower dosage of phenobarbital [47] (although it must be noted this is not a commonly used medication for seizure control/management in middle and high income country medical systems) and having a shorter duration of epilepsy [47]. Aside from seizures, other nervous system conditions, including dementia and Parkinson’s Disease, were also identified. Drowning is both a leading cause of death among those with dementia who die while wandering [36] and a common suicide method for those with dementia [50]. Parkinson’s Disease was reported to impact swimming ability leading to non-fatal drowning [48].

Mental and behavioural conditions was the second most commonly explored category of condition within the included literature. The included literature identified drowning as a popular suicide method for those with schizophrenia [74], psychotic disorders [77] and dementia [50]. Comprehensive psychiatric assessment and management and education in alcohol and substance misuse were recommended as education-based primary prevention strategies for intentional drowning involving mental and behavioural disorders, as well as bystander rescue and CPR training as secondary and tertiary measures [7]. Suicide response training for lifeguard and lifesavers has also been proposed, but not yet implemented or evaluated [68].

Diseases of the circulatory system were highlighted in 30% of included studies. Given ischaemic heart disease remains a leading cause of mortality globally [110], it is unsurprising to see cardiac conditions well represented within the drowning literature. Similarly, physical exercise such as swimming can temporarily increase the risk of aggravating cardiovascular conditions [85]. This is an important challenge, as aquatic exercise can be an effective and low-impact form of exercise, thus improving health and fitness [111]. The prevalence of unknown cardiac disease or cardiac conductivity issues during autopsy was also highlighted [63,83,86,91].

Diseases of the nervous system were also highlighted in the literature. Nervous systems disorders are wide-ranging, and this was reflected in the literature. The conditions that were highlighted appear to reflect those relating to the central nervous system and those that propagate immobility. Although aquatic exercise is often promoted to individuals with these conditions due to the non-weight bearing nature of the exercise, the risk of drowning must be considered. Levels of consciousness and mobility both pose a risk in drowning.

With this exploration of drowning and medical conditions, it was difficult to determine if there was an increased rate of drowning. For future studies, we propose that the studies include the total number of drowning deaths, the total number of people in the population, the population rate of the condition being studied and a relative risk (or similar) for drowning. This would allow future reviews to clearly be able to show the rate of drowning and the rate of drowning in the condition being explored, thus enabling a relative risk to be calculated.

One of the most common drowning prevention recommendations related to supervised bathing or the replacement of bathing with showering for those with diseases of the nervous system, such as seizure disorders [31]. Additionally, it was recommended that care givers of those with diseases of the circulatory system be aware of the drowning risks for those with such conditions, especially in the winter months [53]. For elderly adults with pre-existing medical conditions of any kind, showering with the use of an aid, such as a chair, was also recommended [19]. The majority of proposed drowning prevention encompassed primary drowning prevention strategies; however, many were administrative in nature, reflecting a low level of effectiveness on the hierarchy of control [27]. Additionally, all 25 unique drowning prevention recommendations were proposed only, identifying a knowledge gap regarding the efficacy of interventions based on implementation and evaluation.

Finally, with an aging population and increasing comorbidities comes an increased prescription medical rate, resulting in polypharmacy [112]. Multiple medications can contribute to drowning risk [113]; however, no study to date has examined the complex nature of polypharmacy, pre-existing medical conditions and adult drowning risk. This topic presents an opportunity for future research.

This study is the first to systematically explore the peer-reviewed literature to explore drowning and comorbidities and provides valuable information around conditions increasing drowning risk and research gaps. However, the findings of this study must be considered in light of some limitations. Within the included literature, we did not document if the person who drowned knew they had the particular condition or were treated appropriately for it. Only one included study reported medication levels as a risk factor, exploring phenobarbital levels among epileptics [47]. Secondly, the included studies are where drowning and a particular pre-existing medical condition co-occurred; there did not need to be, nor did we draw, a causal link between drowning and the condition in order for the study to be included in this review. Thirdly, where multiple pre-existing medical conditions are present, we did not examine the attributable drowning risk for individual conditions. All limitations also offer opportunities to strengthen the evidence base around medical conditions and drowning risk in the future.

## 5. Conclusions

Drowning occurs in people with existing medical conditions. This review has highlighted several pre-existing medical conditions that increase drowning risk; however, we also identified numerous research gaps. As we live longer and the proportion of the population with comorbidities increases, there is a need to better quantify the drowning risk associated with pre-existing medical conditions. Future research should include population level studies comparing disease prevalence in the general population to those who drown and better delineate the attributable risk for those with multiple medical conditions. In addition, there is a need for the implementation and evaluation of proposed strategies to reduce drowning burden and the risk associated with pre-existing medical conditions.

## Figures and Tables

**Table 1:** Inclusion and exclusion criteria.

Inclusion	Exclusion
Peer-review literature, 1 January 2005 to 31 October 2021, English, global	Outside date range, non-English language, non-peer-reviewed
Limited to humans	Non-human
Primary research	Protocols, literature reviews
Unintentional drowning	-
Intentional self-harm drowning	Homicide, assault, criminal behaviour
Data from primary analytical studies that include an unintentional drowning or submersion in water AND a medical condition (physiological or psychological) of any description.	Acute conditions such as recent musculoskeletal injuries were excluded (i.e., broken arm during experience due to the drowning event)
Recreational drowning and commercial sub-populations (i.e., fisherman)	-
Medical conditions could be known or unknown by the drowning victim prior to the drowning event (i.e., undiagnosed cardiac arrythmia or epilepsy)	Conditions which were not chronic in nature or occurred as a result of the drowning incident (i.e., pulmonary oedema).
Sample comprised of adult population only or a minimum of 75% of sample or age group could be easily extracted from main data set of relevant studies	Study population aged 0–14 years only, or population aged 15 years or over could not be disaggregated
Case reports included if reports ≥ 6 cases and include a population and provides some indication of risk	Studies reporting < 6 cases.

**Table 2:** Medical condition groupings and examples of included conditions.

Medical Condition Group	Sub-Categorisation	Examples of Included Conditions
Diseases of the circulatory system	Aneurysm and dissections	Aortic aneurysm and dissection
	Atherosclerosis	Atherosclerosis
	Cardiomyopathies	Hypertrophic cardiomyopathy
	Congenital Heart Disease	Congenital coronary arterial anomaly
	Heart Arrythmias	Cardiac arrythmia, Long QT, Cardiac channel mutation, Syncope/Other, Wolf-Parkinson White Syndrome
	Hypertensive Heart Disease	Heart hypertrophy, Left Ventricle Hypertrophy
	Hypertensive Vascular Disease	Presence of cardia and vascular implants and grafts, blood pressure problems
	Ischemic Heart Disease	Coronary artery atherosclerosis; Ischaemic heart disease, coronary artery stenosis, recent myocardial infarction
Diseases of the digestive system		Digestive disease
Diseases of the ear and mastoid		Hearing impairment
Diseases of the eye and adnexa		Blindness; low vision; visual impairment
Diseases of the genitourinary system		Kidney dysfunction
Diseases of the musculoskeletal system and connective tissue		Physical disability
Diseases of the nervous system	Dementia	Dementia
	Seizure disorders	Convulsive epilepsy; epilepsy
	Neurological mobility disorders	Mobility disturbance; Parkinson’s Disease
	Other	Disability of brain lesion; central nervous system disease; nervous disease
	Nervous system (no further breakdown)	-
Diseases of the respiratory system		Asthma; respiratory disease
Endocrine, nutritional, and metabolic diseases		Diabetes; dyslipidemia; obese; overweight; underweight
Mental and behavioural conditions	Anxiety Disorder	Anxiety disorder
	Behavioural Disorder	Somatic disorders, somatic comorbidity, behavioural syndromes associated with psychological disturbances and physical factors
	Cognitive Function	Intellectual disability, mental retardation, senile dementia, disorders of psychological development, organic brain disorders
	Mood Disorder	Depression, bipolar disorder, affective disorder, depressive disorders
	Personality Disorder	Personality disorder
	Psychosexual disorders	Psychosexual disorders
	Psychotic Disorder	Psychiatric disorders, schizophrenia, non-organic psychotic disorder, neurotic disorders
	Substance Abuse Disorders	Alcoholism, drug dependence, poisoning
	Other Disorders	Other disorders (organic disorders)
Neoplasms		Cancer
Symptoms, signs and conditions not elsewhere classified		Pregnancy

**Table 3:** Grouped medical condition by included studies.

Medical Condition Grouped	Number of Studies	% of All Included Studies (n = 83)	Reference(s)
Diseases of the circulatory system	25	30.1	[19,43,46,49,53,54,55,57,63,76,83,84,85,86,87,88,89,90,91,92,93,94,95,96,97,98]
Diseases of the digestive system	1	1.2	[53]
Diseases of the ear and mastoid	1	1.2	[42]
Diseases of the eye and adnexa	2	2.4	[42,99]
Diseases of the genito-urinary system	2	2.4	[42,53]
Diseases of the musculoskeletal system and connective tissue	3	3.6	[19,42,100]
Diseases of the nervous system	32	38.6	[7,11,19,20,30,31,32,33,34,35,36,37,38,39,40,41,42,43,44,45,46,47,48,49,50,51,52,53,54,55,56,57]
Diseases of the respiratory system	5	6.0	[42,43,53,54,76]
Endocrine, nutritional, and metabolic diseases	6	7.2	[11,53,56,63,76,101]
Mental and behavioural conditions	31	37.3	[7,37,42,43,54,56,57,58,59,60,61,62,63,64,65,66,67,68,69,70,71,72,73,74,75,76,77,78,79,80,81,82]
Neoplasms	2	2.4	[53,80]
Symptoms, signs, and conditions not elsewhere classified	1	1.2	[102]

Note: some papers included more than one medical condition, hence the total adds to more than the total number of included studies (83).

**Table 4:** Studies reporting conditions within the diseases of the nervous system category.

Condition	Reference	Country	Study Population	Age Group	Intent	Outcome	% Who Drowned	% Who Drowned with Med Conditions	% Who Drowned with Condition
Dementia	Furumiya et al., 2015 [36]	Japan	Outdoor wandering deaths	70–94 years	U	F		-	42.1%
	Kikuchi et al., 2019 [41]	Japan	Wandering deaths among those with dementia	All ages	U	F		-	11.3%
	Purandare et al., 2009 [50]	UK	Suicide among those with dementia	65+ years	I	F		-	17.8%
	Rowe et al., 2011 [51]	USA	Persons with dementia who go missing	40–95 years	U	F		-	11.4
Seizure disorders	Bain et al., 2018 [11]	Canada	Epilepsy or seizure with suspicion of drowning	12–68 years	U	F			100.0%
	Barooni et al., 2007 [30]	Canada	Epilepsy drowning deaths	0–90 years	U	F	4.1%		100%
	Chang et al., 2012 [33]	Taiwan	Deaths in those with epilepsy	All ages	U	F			0.3%
	Chang et al., 2014 [32]	USA	Epilepsy on death certificates	All ages	U	F			0.21 *
	Cihan et al., 2018 [34]	USA	Epilepsy deaths in water	20–73 years	U	F	-	-	2.7%
	Ding et al., 2013 [35]	China	Epilepsy diagnosis follow-up	10–69 years	U,I,Und	F			1.4%
	Hossain et al., 2017 [37]	Bangladesh	Adult drowning	18+ years	U	F	9.6%		
	Jinda et al., 2019 [38]	Thailand	Seizure related injuries	15+ years	U	F			0.05%
	Kaiboriboon et al., 2014 [39]	USA	Hospitalised epilepsy deaths	18–64 years	U	F			0.4%
	Karlovich et al., 2020 [40]	USA	Deaths in people with history of seizure	18–45 years	U	F			0.2%
	Markarian et al., 2020 [43]	France	Selected patients admitted to ICU for a drowning-related incident	40–74 years	U	F	7.7%	7.8%	-
	Mateen et al., 2012 [44]	Bangladesh	Accidental injury death in people with epilepsy	12–58 years	U	F			83.3%
	Mbizvo et al., 2021 [45]	Scotland	Non SUDEP epilepsy related deaths	≥16 years	U	F			4.4%
	Morris et al., 2016 [46]	South Africa	Bodies retrieved from water and immersion related deaths	18+ years	U	F	3.2%	60.0%	-
	Mu et al., 2011 [47]	China	Death among people with convulsive epilepsy	>15 years	U	F	-	-	1.3%
	Okuda et al. 2015 [49]	USA	Deaths in bathtubs	22–96 year	U,I	F	9.1%	18.8%	-
	Peden et al., 2019 [19]	Australia	Bathtub drownings	65+ years	U	F	6.3%	7.7%	-
	Satoh et al., 2013 [20]	Japan	Sudden deaths in hot bathtubs	8–95 years	U	F	11.1%	-	-
	Sillanpaa et al., 2010 [52]	Finland	Long term mortality among those with childhood-onset epilepsy	1–50 years	U	F	-	-	2.4%
	Suzuki et al., 2015 [53]	Japan	Autopsied bath related deaths	All ages	U	F	2.2%	2.8%	-
	Tellier et al., 2019 [54]	France	Drowning victims along Gironde surf beaches	All ages	U	F	0.2%	2.0%	-
	Yang et al., 2018 [55]	South Korea	Bath-related deaths	18–91 years	U	F	3.5%	4.0%	-
	Youn et al., 2009 [56]	South Korea	OHCA due to drowning admitted to hospital	3–87 years	U,I,Und	F	1.8%	14.3	-
Neurological mobility disorders	Neves et al., 2020 [48]	Portugal & the UK	Patients with Parkinson’s Disease	M = 64 years	U	NF		-	49.1%
	Satoh et al., 2013 [20]	Japan	Sudden deaths in hot bathtubs	8–95 years	U	F	11.1%	-	-
Other	Kim et al., 2021 [42]	South Korea	Deaths of people with a disability	All ages	U	F	-	4.4/100,000 *	-
	Okuda et al., 2015 [49]	USA	Deaths in bathtubs	22–96 year	U,I	F	-	9.4%	-
	Suzuki et al., 2015 [53]	Japan	Autopsied bath related deaths	All ages	U	F	1.1%	-	-
	Yang et al., 2018 [55]	South Korea	Bath-related deaths	18–91 years	U	F	5.3%	6.0%	-
Nervous system (no further breakdown)	Cenderadewi et al., 2019 [7]	Australia	Intentional drowning deaths	All ages	I	F	2.4%	2.4%	-
	Peden et al., 2016 [57]	Australia	River drowning deaths	All ages	U	F	1.7%	4.4%	-

Abbreviations: F = Fatal; I = Intentional; M = mean age; NF = Non-Fatal; OHCA = Out of Hospital Cardiac Arrest; SUDEP = Sudden Unexpected Death in Epilepsy; U = Unintentional; UK = United Kingdom; Und = Undetermined; USA = United States of America; * represents crude mortality rate per 100,000 population not proportion.

**Table 6:** Studies reporting conditions within the diseases of the circulatory system category.

Condition	Reference	Country	Study Population	Age Group	Intent	Outcome	% Who Drowned	% Who Drowned with Med Conditions	% Who Drowned with Condition
Aneurysms and Dissections	Kevekidis et al., 2021 [85]	Greece	Drowning deaths	15–75+ years	U	F	0.4%	0.7%	-
Atherosclerosis	Kevekidis et al., 2021 [85]	Greece	Drowning deaths	15–75+ years	U	F	12.5%	20.0%	-
Cardiomyopathies	Kevekidis et al., 2021 [85]	Greece	Drowning deaths	15–75+ years	U	F	9.6%	14.1%	-
	Yang et al., 2018 [55]	South Korea	Bath-related deaths	18–91 years	U	F	1.8%	2.0%	-
Congenital heart disease	Harris et al., 2017 [83]	USA	Sudden death during sanctioned triathlon	15–80 years	U	F	11.1%	-	-
Heart arrythmias	Guay et al., 2019 [63]	Canada	Bathtub drownings people aged 65+	65+ years	U	F	21.7%	-	-
	Harris et al., 2010 [84] Harris et al., 2017 [83]	USA	Sudden death in USA Triathlon sanctioned events	-	U	F	11.1%	-	-
	Lippmann et al. 2021 [86]	New Zealand	Breath-hold diving fatalities	24–70 years	U	F	20.7%	-	-
	Peden et al., 2019 [19]	Australia	Bathtub drownings	65–85+ years	U	F	15.6%	19.2%	-
	Tester et al., 2011 [91]	USA	Unexplained drowning victims referred for a cardiac channel molecular autopsy	3.5–69 years	U	F	22.9%	-	-
	Walker et al., 2006 [95]	Australia	Diving-related fatalities	21–81 years	U	F	22.2%	-	-
	Walker et al., 2009 [96]	Australia	Diving-related fatalities	20–65 years	U	F	11.1%	-	-
Hypertensive heart disease	Harris et al., 2010 [84]	USA	Competitors in USA Triathlon sanctioned events	-	U	F	66.7%	-	-
	Harris et al., 2017 [83]	USA	Sudden death during sanctioned triathlon	15–80 years	U	F	66.7%	-	-
Hypertensive vascular disease	Guay et al., 2019 [63]	Canada	Bathtub drownings people aged 65+	65+ years	U	F	15.2%	-	-
	Kevekidis et al., 2021 [85]	Greece	Drowning deaths	15–75+ years	U	F	2.1%	3.3%	-
	Schneppe et al., 2021 [90]	Germany	Deaths in water	1–90 years	I, U	F	14.3%	38.3%	-
Ischaemic heart disease	Kevekidis et al., 2021 [85]	Greece	Drowning deaths	15–75+ years	U	F	87.9%	--	-
	Papadodima et al., 2007 [89]	Greece	Drowning victims	<15–74+ years	U	F	51.8%	-	-
	Schneppe et al., 2021 [90]	Germany	Deaths in water	1–90 years	I, U	F	23.0%	61.7%	-
	Suzuki et al., 2015 [53]	Japan	Autopsied bath related deaths	0–90+ years	U	F	34.2%	43.2%	-
	Yang et al., 2018 [55]	South Korea	Bath-related deaths	18–91 years	U	F	73.7%	84.0%	-
All cardiovascular conditions	Claesson et al., 2013 [97]	Sweden	Swedish National Board of Forensic Medicine autopsied drowning cases	22–71 years	I, U, Und	F	10.1%	-	-
	Guay et al., 2019 [63]	Canada	Bathtub drownings people aged 65+	65+ years	U	F	6.5%	30.0%	-
	Harris et al., 2010 [84]	USA	Competitors in USA Triathlon sanctioned events	-	U	F	77.8%	-	-
	Harris et al., 2017 [83]	USA	Sudden death during sanctioned triathlon	15–80 years	U	F	77.8%	-	-
	Kevekidis et al., 2021 [85]	Greece	Drowning deaths	15–75+ years	U	F	62.5%	-	
	Lippmann et al. 2021 [86]	New Zealand	Breath-hold diving fatalities	24–70 years	U	F	34.5%	-	-
	Markarian et al., 2020 [43]	France	Selected patients admitted to ICU for a drowning-related incident	40–74 years	I	F	35.6%	26.0%	--
	Mishima et al., 2018 [87]	Japan	Bath-related deaths	34–92 years	U	F	24.4%	28.6%	-
	Morgan et al., 2008 [88]	Australia	Surf beach swimmers and surfers	13–86 years	U	F	26.4%	87.2%	-
	Morris et al., 2016 [46]	South Africa	Bodies retrieved from water and immersion related deaths	18+ years	U	F	4.8%	40.0%	-
	Okuda et al., 2015 [49]	USA	Deaths in bathtubs	22–96 year	U,I	F	30.3%	62.5%	-
	Peden et al., 2016 [57]	Australia	River drowning deaths	0–75+ years	U	F	5.6%	14.8%	-
	Peden et al., 2019 [19]	Australia	Bathtub drownings	65–85+ years	U	F	43.8%	53.8%	-
	Reizine et al., 2021 [76]	France	Death after non-fatal drowning in fresh and sea water	M age = 68 years	I,U	F	10.7%	14.5%	-
	Suzuki et al., 2015 [53]	Japan	Autopsied bath related deaths	0–90+ years	U	F	50.9%	64.4%	-
	Tellier et al., 2019 [54]	France	Drowning victims along Gironde surf beaches	0–65+ years	U	F	3.0%	34.7%	-
	Tikka et al., 2021 [92]	Finland	Land motor traffic crash related drownings	M age = 34.7 years	I,U	F	9.0%	-	-
	Vinkel et al., 2016 [94]	Denmark	Diving-related fatalities	21–59 years	U	F	20.8%	-	-
	Yang et al., 2018 [55]	South Korea	Bath-related deaths	18–91 years	U	F	-	86.0%	-

Abbreviations: F = Fatal; I = Intentional; U = Unintentional; Und = Undetermined; USA = United States of America.

**Table 8:** Drowning prevention strategies documented in included literature by medical condition.

Prevention Strategy Coded	Prevention Strategy Free Text	Medical Condition Category	Drowning Intent	Primary, Secondary or Tertiary	Proposed (P), Implemented (I) or Evaluated (E)	Hierarchy of Control	Reference
Education	Counselling regarding drowning prevention for people with epilepsy	Nervous system	Unintentional	Primary	P	Administrative	Bain et al. 2018 [11]
Education	Routinely warn people with epilepsy about the potential for drowning while bathing	Nervous system	Unintentional	Primary	P	Administrative	Bowman et al. 2010 [31]
Education	Encourage showering alternatively	Nervous system	Unintentional	Primary	P	Substitution	Bowman et al. 2010 [31]
Education	Children and those who require dependent care, who may not be capable of showering, should not be left unattended in a bathtub	Nervous system	Unintentional	Primary	P	Administrative	Bowman e al 2010 [31]
Education	Promote impact of alcohol and substance misuse	Mental and behavioural	Intentional	Primary	P	Administrative	Cenderadewi et al. 2019 [7]
Education	Supervision and specific bathing precautions could be effective prevention strategies	Nervous system	Unintentional	Primary	P	Administrative	Cihan et al., 2018 [34]
Education	Policymakers and healthcare professionals should increase public awareness that people whose families consider their cognitive function normal or normal for their age can go missing	Nervous system	Unintentional	Secondary	P	Administrative	Kikuchi et al., 2019 [41]
Education	Water safety programs for people of all ages with epilepsy, appropriate for level of ability	Nervous system	Unintentional	Primary	P	Administrative	Mateen et al., 2012 [44]
Education	Educating people with epilepsy and their carers of the risks of drowning	Nervous system	Unintentional	Primary	P	Administrative	Mu et al., 2011 [47]
Education	Alert those with Parkinson’s Disease to the potential risks associated with swimming and the need to understand the disease-related features that contribute to the changes in swimming performance	Nervous system	Unintentional	Primary	P	Administrative	Neves et al., 2020 [48]
Education	Increased GP and carer awareness of role of medical conditions and bathtub drowning risk	All pre-existing medical conditions	Unintentional	Primary	P	Administrative	Peden et al., 2019 [19]
Education	Showering is a safer solution especially where showering aids such as chairs are used	All pre-existing medical conditions	Unintentional	Primary	P	Substitution	Peden et al., 2019 [19]
Education	Family members should pay attention to elderly people who have circulatory diseases during bathing, particularly in winter	Circulatory system	Unintentional	Primary	P	Administrative	Suzuki et al., 2015 [53]
Guidelines	Prevention and treatment guidelines developed	Nervous system	Unintentional	Primary	P	Administrative	Mu et al., 2011 [47]
Policy	Prevent dementia patients who live alone from going missing and ensure their absence is noticed immediately	Nervous system	Unintentional	Primary	P	Administrative	Kikuchi et al., 2019 [41]
Policy	Local governments should appeal to inhabitants for cooperation with search activities	Nervous system	Unintentional	Secondary	P	Administrative	Kikuchi et al., 2019 [41]
Policy	Local governments, police stations and MESN should be prepared to initiate search activities immediately	Nervous system	Unintentional	Secondary	P	Administrative	Kikuchi et al., 2019 [41]
Testing	Patients with cardiovascular disease, and particularly those with infarctions, should undergo special testing (e.g., Holter monitoring during swimming)	Circulatory system	Unintentional	Primary	P	Administrative	Papadodima et al., 2007 [89]
Testing	A medical check-up for those who go diving	Circulatory system	Unintentional	Primary	P	Administrative	Peden et al., 2016 [57]
Testing (genetic)	Testing considered in post-mortem evaluation of unexplained drowning, especially if positive personal or family history is elicited to identify cardiac channel mutation	Circulatory system	Unintentional	Primary	P	Administrative	Tester et al., 2011 [91]
Testing	Over 45 years of age, divers and snorkelers should have their cardiovascular health periodically assessed by a dive doctor, preferably well aware of the cardiovascular stressors associated with diving and snorkelling	Circulatory system	Unintentional	Primary	P	Administrative	Walker et al., 2009 [96]
Training	Bystander rescue and CPR training	Mental and behavioural	Intentional	Secondary (rescue) Tertiary (CPR)	P	Administrative	Cenderadewi et al. 2019 [7]
Training	Development of suicide-response training by surf lifesaving volunteers	Mental and behavioural	Intentional	Primary	P	Administrative	Lawes et al., 2021 [68]
Treatment	Design a comprehensive psychiatric assessment and management plan, by promoting identification, treatment and follow-up of individuals with psychiatric conditions	Mental and behavioural	Intentional	Primary	P	Administrative	Cenderadewi et al. 2019 [7]
Treatment	Intensified aftercare is warranted after suicide attempts	Mental and behavioural	Intentional	Primary	P	Administrative	Runeson et al., 2010 [77]

Abbreviations: CPR = Cardio-pulmonary Resuscitation; GP = General Practitioner; MESN = Missing Elderly Search Network.

## Data Availability

Not applicable.

## Appendix A

**Table A1:** Databases and Search Terms Used.

Search Number	Search Term
**MEDLINE (Ovid)**	
1	exp Drowning/3
2	exp Water Sports/
3	exp *Immersion/
4	lakes/ or exp “oceans and seas”/ or ponds/ or rivers/ or dams/
5	bathing beaches/or swimming pools/
6	1 or 2 or 3 or 4 or 5
7	exp Death/
8	exp Mortality/
9	exp Morbidity/
10	7 or 8 or 9
11	exp Chronic Disease/
12	exp Epilepsy/
13	exp Diabetes Mellitus/
14	exp Mental Disorders/
15	exp respiratory tract infections/or exp neoplasms/or exp musculoskeletal diseases/or exp digestive system diseases/or exp stomatognathic diseases/ or exp respiratory tract diseases/or exp otorhinolaryngologic diseases/or exp nervous system diseases/or exp eye diseases/or exp male urogenital diseases/or exp “female urogenital diseases and pregnancy complications”/or exp cardiovascular diseases/or exp “hemic and lymphatic diseases”/or exp “congenital, hereditary, and neonatal diseases and abnormalities”/or exp “skin and connective tissue diseases”/or exp “nutritional and metabolic diseases”/or exp endocrine system diseases/or exp immune system diseases/or exp “disorders of environmental origin”/or exp occupational diseases/or exp chemically-induced disorders/or exp “wounds and injuries”/
16	11 or 12 or 13 or 14 or 15
17	6 and 16
18	10 and 17
19	exp adult/
20	18 and 19
21	limit 20 to (english language and humans and yr = “2005–2021”)
**PUBMED**	
	**((drown* OR submer*) AND (death* OR mortality OR morbidity)) AND ((“chronic disease*”) OR (“chronic illness*”) OR (epilep*) OR (seizure*) (arrest*) OR (cardiac*) OR (cardio*) OR (pulmon*) OR (asystole*) OR (heart*) OR (lung*) OR (diabet*) OR (respir*) OR (neoplasm*) OR (cancer*) OR (musculoskeletal*) OR (digest*) OR (stomatognathic*) OR (lymphat*) OR (vascul*) OR (congenital*) OR (hereditary*) OR (metabol*) OR (endocrin*) OR (immun*) OR (liver*) OR (arrythmia*) OR (“multiple sclerosis”) OR (motor*) OR (dementia*) OR (Alzheimer*) OR (parkinson*) OR (nervous*) OR (nerve*) OR (neuro*) OR (Amyotrophic Lateral Sclerosis*) OR (autism*) OR (addict*) OR (mental*) OR (psych*) OR (“medical condition*)) AND ((humans[Filter]) AND (english[Filter]) AND (alladult[Filter]) AND (2005:2020[pdat])) Filters: Humans, English, Adult: 19+ years** (((“drown*”[All Fields] OR “submer*”[All Fields]) AND ((“death*”[All Fields] OR (((“mortality”[MeSH Terms] OR “mortality”[All Fields]) OR “mortalities”[All Fields]) OR “mortality”[MeSH Subheading])) OR ((((((“epidemiology”[MeSH Subheading] OR “epidemiology”[All Fields]) OR “morbidity”[All Fields]) OR “morbidity”[MeSH Terms]) OR “morbid”[All Fields]) OR “morbidities”[All Fields]) OR “morbids”[All Fields]))) AND ((((((((((((((((((((((((((((((((((((((((“chronic disease*”[All Fields] OR “chronic illness*”[All Fields]) OR “epilep*”[All Fields]) OR “seizure*”[All Fields]) AND “arrest*”[All Fields]) OR “cardiac*”[All Fields]) OR “cardio*”[All Fields]) OR “pulmon*”[All Fields]) OR “asystole*”[All Fields]) OR “heart*”[All Fields]) OR “lung*”[All Fields]) OR “diabet*”[All Fields]) OR “respir*”[All Fields]) OR “neoplasm*”[All Fields]) OR “cancer*”[All Fields]) OR “musculoskeletal*”[All Fields]) OR “digest*”[All Fields]) OR “stomatognathic*”[All Fields]) OR “lymphat*”[All Fields]) OR “vascul*”[All Fields]) OR “congenital*”[All Fields]) OR “hereditary*”[All Fields]) OR “metabol*”[All Fields]) OR “endocrin*”[All Fields]) OR “immun*”[All Fields]) OR “liver*”[All Fields]) OR “arrythmia*”[All Fields]) OR “multiple sclerosis”[All Fields]) OR “motor*”[All Fields]) OR “dementia*”[All Fields]) OR “alzheimer*”[All Fields]) OR “parkinson*”[All Fields]) OR “nervous*”[All Fields]) OR “nerve*”[All Fields]) OR “neuro*”[All Fields]) OR (“Amyotrophic”[All Fields] AND (((((((((((((((((((“functional laterality”[MeSH Terms] OR (“functional”[All Fields] AND “laterality”[All Fields])) OR “functional laterality”[All Fields]) OR “laterality”[All Fields]) OR “lateral”[All Fields]) OR “lateralisation”[All Fields]) OR “lateralisations”[All Fields]) OR “lateralise”[All Fields]) OR “lateralised”[All Fields]) OR “lateralises”[All Fields]) OR “lateralising”[All Fields]) OR “lateralities”[All Fields]) OR “lateralization”[All Fields]) OR “lateralizations”[All Fields]) OR “lateralize”[All Fields]) OR “lateralized”[All Fields]) OR “lateralizes”[All Fields]) OR “lateralizing”[All Fields]) OR “laterally”[All Fields]) OR “laterals”[All Fields]) AND “sclerosis*”[All Fields])) OR “autism*”[All Fields]) OR “addict*”[All Fields]) OR “mental*”[All Fields]) OR “psych*”[All Fields]) OR (((((((((((((((((((“medic”[All Fields] OR “medical”[All Fields]) OR “medicalization”[MeSH Terms]) OR “medicalization”[All Fields]) OR “medicalizations”[All Fields]) OR “medicalize”[All Fields]) OR “medicalized”[All Fields]) OR “medicalizes”[All Fields]) OR “medicalizing”[All Fields]) OR “medically”[All Fields]) OR “medicals”[All Fields]) OR “medicated”[All Fields]) OR “medication s”[All Fields]) OR “medics”[All Fields]) OR “pharmaceutical preparations”[MeSH Terms]) OR (“pharmaceutical”[All Fields] AND “preparations”[All Fields])) OR “pharmaceutical preparations”[All Fields]) OR “medication”[All Fields]) OR “medications”[All Fields]) AND “condition*”[All Fields]))) AND (((“humans”[MeSH Terms] AND “english”[Language]) AND “adult”[MeSH Terms]) AND 2005/1/1:2021/10/31[Date-Publication])
**SCOPUS**	
	((TITLE-ABS-KEY (*drown**) OR TITLE-ABS-KEY (*submer**)) AND (TITLE-ABS-KEY (*death**) OR TITLE-ABS-KEY (*mortality**) OR TITLE-ABS-KEY (*morbidity**))) AND (TITLE-ABS-KEY (*“chronic disease*”*) OR TITLE-ABS-KEY (*“chronic illness*”*) OR TITLE-ABS-KEY (*epilep**) OR TITLE-ABS-KEY (*seizure**) OR TITLE-ABS-KEY (*arrest**) OR TITLE-ABS-KEY (*cardiac**) OR TITLE-ABS-KEY (*cardio**) OR TITLE-ABS-KEY (*pulmon**) OR TITLE-ABS-KEY (*asystole**) OR TITLE-ABS-KEY (*heart**) OR TITLE-ABS-KEY (*lung**) OR TITLE-ABS-KEY (*diabet**) OR TITLE-ABS-KEY (*respir**) OR TITLE-ABS-KEY (*neoplasm**) OR TITLE-ABS-KEY (*cancer**) OR TITLE-ABS-KEY (*musculoskeletal**) OR TITLE-ABS-KEY (*digest**) OR TITLE-ABS-KEY (*stomatognathic**) OR TITLE-ABS-KEY (*lymphat**) OR TITLE-ABS-KEY (*vascul**) OR TITLE-ABS-KEY (*congenital**) OR TITLE-ABS-KEY (*hereditary**) OR TITLE-ABS-KEY (*metabol**) OR TITLE-ABS-KEY (*endocrin**) OR TITLE-ABS-KEY (*immun**) OR TITLE-ABS-KEY (*liver**) OR TITLE-ABS-KEY (*arrythmia**) OR TITLE-ABS-KEY (*“multiple sclerosis”*) OR TITLE-ABS-KEY (*motor**) OR TITLE-ABS-KEY (*dementia**) OR TITLE-ABS-KEY (*alzheimer**) OR TITLE-ABS-KEY (*parkinson**) OR TITLE-ABS-KEY (*nervous**) OR TITLE-ABS-KEY (*nerve**) OR TITLE-ABS-KEY (*neuro**) OR TITLE-ABS-KEY (*amyotrophic* AND *lateral* AND *sclerosis**) OR TITLE-ABS-KEY (*autism**) OR TITLE-ABS-KEY (*addict**) OR TITLE-ABS-KEY (*mental**) OR TITLE-ABS-KEY (*psych**) OR TITLE-ABS-KEY (*“medical condition*”*)) AND TITLE-ABS-KEY (*adult**) AND TITLE-ABS-KEY (*human**) AND (LIMIT-TO (PUBYEAR, *2021*) OR LIMIT-TO (PUBYEAR, *2020*) OR LIMIT-TO (PUBYEAR, *2019*) OR LIMIT-TO (PUBYEAR, *2018*) OR LIMIT-TO (PUBYEAR, *2017*) OR LIMIT-TO (PUBYEAR, *2016*) OR LIMIT-TO (PUBYEAR, *2015*) OR LIMIT-TO (PUBYEAR, *2014*) OR LIMIT-TO (PUBYEAR, *2013*) OR LIMIT-TO (PUBYEAR, *2012*) OR LIMIT-TO (PUBYEAR, *2011*) OR LIMIT-TO (PUBYEAR, *2010*) OR LIMIT-TO (PUBYEAR, *2009*) OR LIMIT-TO (PUBYEAR, *2008*) OR LIMIT-TO (PUBYEAR, *2007*) OR LIMIT-TO (PUBYEAR, *2006*) OR LIMIT-TO (PUBYEAR, *2005*)) AND (LIMIT-TO (EXACTKEYWORD, “*Human*”) OR LIMIT-TO (EXACTKEYWORD, *“Adult”*) OR LIMIT-TO (EXACTKEYWORD, *“Humans”*)) AND (LIMIT-TO (LANGUAGE, *“English”*))
**PsycINFO (ProQuest)**	
	(drown* OR submers*) AND (death* OR mortality OR morbidity) AND ((chronic disease*) OR (chronic illness*) OR (epilep*) OR (seizure*) (arrest*) OR (cardiac*) OR (cardio*) OR (pulmon*) OR (asystole*) OR (heart*) OR (lung*) OR (diabet*) OR (respir*) OR (neoplasm*) OR (cancer*) OR (musculoskeletal*) OR (digest*) OR (stomatognathic*) OR (lymphat*) OR (vascul*) OR (congenital*) OR (hereditary*) OR (metabol*) OR (endocrin*) OR (immun*) OR (liver*) OR (arrythmia*) OR (multiple sclerosis) OR (motor*) OR (dementia*) OR (Alzheimer*) OR (parkinson*) OR (nervous*) OR (nerve*) OR (neuro*) OR (Amyotrophic Lateral Sclerosis*) OR (autism*) OR (addict*) OR (mental*) OR (psych*)) Date: After 01 January 2005 Language English Age group Adulthood (18 Yrs & Older) Population Human
**SPORTSDiscus**	
	(drown* OR submers* OR river* OR lake* OR shower* OR bath* OR dam* OR beach* OR pool* OR pond* OR ocean) AND (death* OR mortality OR morbidity) AND ((chronic disease*) OR (chronic illness*) OR (epilep*) OR (seizure*) (arrest*) OR (cardiac*) OR (cardio*) OR (pulmon*) OR (asystole*) OR (heart*) OR (lung*) OR (diabet*) OR (respir*) OR (neoplasm*) OR (cancer*) OR (musculoskeletal*) OR (digest*) OR (stomatognathic*) OR (lymphat*) OR (vascul*) OR (congenital*) OR (hereditary*) OR (metabol*) OR (endocrin*) OR (immun*) OR (liver*) OR (arrythmia*) OR (multiple sclerosis) OR (motor*) OR (dementia*) OR (Alzheimer*) OR (parkinson*) OR (nervous*) OR (nerve*) OR (neuro*) OR (Amyotrophic Lateral Sclerosis*) OR (autism*) OR (addict*) OR (mental*) OR (psych*)) **Limiters**—Published Date: 20050101–20211031; Peer Reviewed; Language: English **Expanders**—Apply equivalent subjects **Search modes**—Boolean/Phrase
**EMBASE (Ovid)**	
1	exp Drowning/3
2	exp Water Sports/
3	exp *Immersion/
4	lakes/or exp “oceans and seas”/or ponds/or rivers/or dams/
5	bathing beaches/ or swimming pools/
6	1 or 2 or 3 or 4 or 5
7	exp Death/
8	exp Mortality/
9	exp Morbidity/
10	7 or 8 or 9
11	exp Chronic Disease/
12	exp Epilepsy/
13	exp Diabetes Mellitus/
14	exp Mental Disorders/
15	exp respiratory tract infections/or exp neoplasms/or exp musculoskeletal diseases/or exp digestive system diseases/or exp stomatognathic diseases/or exp respiratory tract diseases/or exp otorhinolaryngologic diseases/or exp nervous system diseases/or exp eye diseases/or exp male urogenital diseases/or exp “female urogenital diseases and pregnancy complications”/or exp cardiovascular diseases/or exp “hemic and lymphatic diseases”/or exp “congenital, hereditary, and neonatal diseases and abnormalities”/or exp “skin and connective tissue diseases”/or exp “nutritional and metabolic diseases”/or exp endocrine system diseases/or exp immune system diseases/or exp “disorders of environmental origin”/or exp occupational diseases/or exp chemically-induced disorders/or exp “wounds and injuries”/
16	11 or 12 or 13 or 14 or 15
17	6 and 16
18	10 and 17
19	exp adult/
20	18 and 19
21	limit 20 to (english language and humans and yr = “2005–2021”)

## Appendix B

**Table A2:** Characteristics of Included Studies (n = 83).

Reference	Study Period	Study County	World Bank Region	Income Level	Evidence Level	Study Population	Age Group	Drowning Outcome		Drowning Intent		
								F	NF	U	I	Und
Aaltonen et al., 2019 [58]	1991–2011	Finland	Europe and Central Asia	HIC	IV	Suicide after first lifetime psychiatric hospitalisation for depression	18+	X			X	
Ahlm et al., 2015 [59]	1992–2009	Sweden	Europe and Central Asia	HIC	III-3	Total population drowned in Sweden	16–85	X			X	
Bain et al., 2018 [11]	2014–2016	Canada	North America	HIC	III-3	Epilepsy or seizure with suspicion of drowning	12–68	X		X		
Barooni et al., 2007 [30]	2004	Canada	North America	HIC	III-3	Epilepsy population deaths	0–90	X		X		
Bjorkenstam et al., 2016 [60]	1987–2013	Sweden	Europe and Central Asia	HIC	IV	Population with personality disorders	15–64	X			X	
Bowman et al., 2010 [31]	1999–2005	USA	North America	HIC	III-3	Patients with epilepsy	0–64	X		X	X	X
Cenderadewi et al., 2019 [7]	2006–2014	Australia	East Asia and Pacific	HIC	III-3	All age intentional drowning deaths	0–75+	X			X	
Chang et al., 2012 [33]	1989–2008	Taiwan	East Asia and Pacific	HIC	III-3	Deaths with epilepsy	0–70+	X		X		
Chang et al., 2014 [32]	1981–2010	USA	North America	HIC	III-3	Mentions of epilepsy on death certificate	0–65+	X		X		
Cihan et al., 2018 [34]	2000–2016	USA	North America	HIC	III-3	Epilepsy deaths in water	20–73	X		X		
Claesson et al., 2013 [97]	2002–2010	Sweden	Europe and Central Asia	HIC	III-3	Autopsied drowning cases (Swedish National Board of Forensic Medicine)	22–71	X		X	X	X
Clemens et al., 2016 [98]	2008–2012	Canada	North America	HIC	III-3	Drowning incidents in Canada	15–65+	X		X		
Ding et al., 2013 [35]	2000–2004	China	East Asia and Pacific	UMIC	III-3	Diagnosis of epilepsy at primary health centre	10–69	X		X	X	X
Fang et al., 2015 [61]	2010–2014	China	East Asia and Pacific	UMIC	III-3	Individuals with psychiatric disorder who committed suicide by drowning	10–89	X			X	
Flaig et al., 2013 [62]	2006–2010	Germany	Europe and Central Asia	HIC	III-3	Autopsied non-natural deaths	18–96	X			X	
Furumiya et al., 2015 [36]	2003–2013	Japan	East Asia and Pacific	HIC	IV	Elderly persons with dementia who died outdoors after wandering	70–94	X		X		
Guay et al., 2019 [63]	2005–2014	Canada	North America	HIC	III-3	Bathtub drownings in the province of Quebec	65+	X		X		
Haines et al., 2010 [64]	-	Australia	East Asia and Pacific	HIC	III-3	Completed suicides in Tasmania	-	X			X	
Harris et al., 2010 [84]	2006	USA	North America	HIC	III-3	Sanctioned triathlete events	-	X		X		
Harris et al., 2017 [83]	1985–2016	USA	North America	HIC	IV	Sanctioned triathlete events	15–80	X		X		
Hong et al., 2013 [103]	2004	South Korea	East Asia and Pacific	HIC	III-3	Korea National Hospital Discharge Survey	0–65+	X		X		
Hossain et al., 2017 [37]	2003	Bangladesh	South Asia	LMIC	III-3	Adult drowning	18+	X		X		
Hunt et al., 2006 [65]	1996–2000	United Kingdom	Europe and Central Asia	HIC	III-3	Sample of cases of suicide in England and Wales with recent (<1 year) contact with mental health services	0–75+	X			X	
Jinda et al., 2019 [38]	2004–2013	Thailand	East Asia and Pacific	UMIC	III-3	Seizure-related injuries	15–80+	X		X		
Kaiboriboon, et al. 2014 [39]	1992–2008	USA	North America	HIC	III-3	Hospitalised epilepsy deaths	18–64	X		X		
Karlovich et al., 2020 [40]	2014–2017	USA	North America	HIC	IV	Decedents with a history of seizure or epilepsy	18–45	X		X		
Kevrekidis et al., 2021 [85]	2009–2018	Greece	Europe and Central Asia	HIC	III-3	Retrospective case–control study of drowning deaths	15–75+	X		X		
Kielty et al., 2015 [66]	2006–2016	Ireland	Europe and Central Asia	HIC	III-3	Probable suicide deaths	18–55+	X			X	
Kikuchi e al, 2019 [41]	2015	Japan	East Asia and Pacific	HIC	IV	Dementia patients missing after wandering	<65–95+	X		X		
Kim et al., 2021 [42]	2008–2017	South Korea	East Asia and Pacific	HIC	III-3	Deaths of people with disabilities registered at Ministry of Health and Welfare	0–80+	X		X		
Kong et al., 2021 [102]	2009–2019	Hunan, China	East Asia and Pacific	UMIC	III-3	Accidental deaths during pregnancy and puerperium	-	X		X		
Koo et al., 2019 [67]	2000–2013	Australia	East Asia and Pacific	HIC	III-3	Cases from the Queensland Suicide Register	65+	X			X	
Kotsiou et al., 2014 [104]	2012–2013	Greece	Europe and Central Asia	HIC	III-3	Drowning hospitalisations	1888	X		X		
Kumar et al. 2017 [72]	2012–2014	India	South Asia	LMIC	IV	Attempted suicides in psychiatric consultation	10–50		X		X	
Lawes et al., 2021 [68]	2005–2019	Australia	East Asia and Pacific	HIC	III-3	Suicidal deaths along the Australian coast	18–70+	X			X	
Lawes et al., 2021 [105]	2004–2019	Australia	East Asia and Pacific	HIC	III-3	Males (15–34 years) were compared with other adults (15 years and older)	15+	X		X		
Lee et al., 2019 [69]	1997–2016	South Korea	East Asia and Pacific	HIC	III-3	Elderly drowning patients	18–65+	X		X	X	X
Lippmann et al., 2021 [86]	2007–2016	New Zealand	East Asia and Pacific	HIC	III-3	Diving fatalities	24–70	X		X		
Ljusic et al., 2018 [70]	2001–2010	Republic of Serbia	Europe and Central Asia	UMIC	III-3	Suicide with mental disorders, somatic disorders or without registered disorder	-	X			X	
Lofman et al., 2011 [100]	1988–2007	Finland	Europe and Central Asia	HIC	III-3	Suicides in the province of Oulu in Northern Finland	0–65+	X			X	
Lunetta et al., 2020 [106]	1971–2013	Finland	Europe and Central Asia	HIC	III-3	Land motor vehicle drowning	0–99+	X		X		
Maity et al., 2020 [71]	2012–2013	India	South Asia	LMIC	III-3	Drowning deaths	0–70	X				X
Markarian et al., 2020 [43]	2014–2017	France	Europe and Central Asia	HIC	III-3	Adult (>18 years of age) ICU admissions	40–74	X		X		
Mateen et al., 2012 [44]	2005–2008	Bangladesh	South Asia	LMIC	III-3	Accidental injury death in people with epilepsy	12–58	X		X		
Mbizvo et al., 2021 [45]	2009–2016	Scotland	Europe and Central Asia	HIC	III-3	Non SUDEP epilepsy related deaths	≥16	X		X		
Meyer-Rochow et al., 2015 [99]	1982–2011	Finland	Europe and Central Asia	HIC	III-3	Suicides among visually impaired persons	20–65+	X			X	
Mishima et al., 2018 [87]	2016	Japan	East Asia and Pacific	HIC	IV	Bath-related deaths	34–92	X			X	
Morgan et al., 2008 [88]	2001–2005	Australia	East Asia and Pacific	HIC	III-3	Surf beach swimmers and surfers	13–86	X		X		
Morris et al., 2016 [46]	2002–2011	South Africa	Sub-Saharan Africa	UMIC	III-3	Bodies retrieved from water and immersion-related deaths in Pretoria	18+	X		X		
Mu et al., 2011 [47]	2005–2009	China	East Asia and Pacific	UMIC	IV	Death among people with convulsive epilepsy in rural West China	<15–66+	X		X		
Neves et al., 2020 [48]	-	Portugal & UK	Europe and Central Asia	HIC	IV	Patients with Parkinson’s Disease	Mean = 64		X	X		
Nishida et al., 2015 [73]	2006–2013	Japan	East Asia and Pacific	HIC	IV	Patients diagnosed with early post stroke depression who died	65–94	X			X	
Okuda et al., 2015 [49]	2003–2013	USA	North America	HIC	IV	Deaths in bathtubs	22–96	X		X	X	
Pan et al., 2021 [74]	2001–2016	Taiwan	East Asia and Pacific	HIC	III-3	Suicide mortality in patients with schizophrenia	<20− ≥80+	X			X	
Papadodima et al., 2007 [89]	1997–2004	Greece	Europe and Central Asia	HIC	III-3	Drowning deaths	<15–74+	X		X		
Park et al., 2013 [75]	1995–2006	South Korea	East Asia and Pacific	HIC	III-3	Psychiatric patients who suicide	10–70+	X			X	
Peden et al., 2019 [108]	2005–2014	Australia/Canada/NZ	-	HIC	III-3	Residents	0–65+	X		X		X
Peden et al., 2016 [57]	2002–2012	Australia	East Asia and Pacific	HIC	III-3	River drowning	0–75+	X		X		X
Peden et al., 2016 [107]	2002–2012	Australia	East Asia and Pacific	HIC	III-3	International tourists to Australia	0–55+	X		X		X
Peden et al., 2019 [19]	2002–2012	Australia	East Asia and Pacific	HIC	III-3	Bathtub drowning	65–85+	X		X		X
Purandare et al., 2009 [50]	1996–2004	United Kingdom	Europe and Central Asia	HIC	III-3	Suicide among those with dementia	65+	X			X	
Reizine et al., 2021 [76]	2013–2020	France	Europe and Central Asia	HIC	IV	Drowning in fresh and sea water	Mean 68	X	X	X	X	
Rowe et al., 2011 [51]	2003–2008	USA	North America	HIC	IV	Persons with dementia who go missing	40–95	X		X		
Runeson et al., 2010 [77]	1973–1982	Sweden	Europe and Central Asia	HIC	III-3	Completed suicides among those treated for attempted suicide	10+	X			X	
Satoh et al., 2013 [20]	1998–2007	Japan	East Asia and Pacific	HIC	III-3	Sudden deaths in hot bathtubs	8–95	X		X		
Schaffer et al., 2014 [78]	1998–2010	Canada	North America	HIC	III-3	Suicide in bipolar disorder	<24–65+	X			X	
Schneppe et al., 2021 [90]	1997–2017	Germany	Europe and Central Asia	HIC	IV	Deaths in water	1–90	X		X	X	
Selvaraj et al., 2020 [79]	2017–2018	India	South Asia	LMIC	IV	Drowning in Madurai Region	0–70+	X		X	X	
Sillanpaa et al., 2010 [52]	1964–2002	Finland	Europe and Central Asia	HIC	IV	Long-term mortality among those with childhood-onset epilepsy	1–50	X		X		
Stemberga et al., 2010 [80]	1981–2005	Croatia	Europe and Central Asia	HIC	III-3	Suicidal drowning deaths	23–86	X			X	
Stephenson et al., 2020 [81]	1988–2017	Australia	East Asia and Pacific	HIC	IV	Urban section of the River Torrens	18–76	X		X	X	
Suzuki et al., 2015 [53]	2009–2011	Japan	East Asia and Pacific	HIC	III-3	Autopsied bath-related deaths	0–90+	X		X		
Tellier et al., 2019 [54]	2011–2016	France	Europe and Central Asia	HIC	III-3	Gironde surf beaches	0–65+	X		X		
Tester et al., 2011 [91]	1998–2010	USA	North America	HIC	IV	Unexplained drowning victims referred for a cardiac channel molecular autopsy	3.5–69	X		X		
Tikka et al., 2021 [92]	1975–2015	Finland	Europe and Central Asia	HIC	IV	Land motor traffic crash related drownings	Mean 34.7	X		X	X	
Tzimas et al., 2016 [93]	2003–2011	Germany	Europe and Central Asia	HIC	IV	Water-related deaths with adequate genetic material for DNA analysis	20–50	X		X		
Vinkel et al., 2016 [94]	1999–2012	Denmark	Europe and Central Asia	HIC	III-3	Diving-related fatalities	21–59	X		X		
Walker et al., 2006 [95]	2001	Australia	East Asia and Pacific	HIC	III-3	Diving-related fatalities	21–81	X		X		
Walker et al., 2009 [96]	2004	Australia	East Asia and Pacific	HIC	III-3	Diving-related fatalities	20–65	X		X		
Williams et al., 2018 [82]	2013–2017	USA	North America	HIC	III-3	Actively serving US armed forces	<20–40 + years	X	X	X		
Wingren et al., 2016 [101]	1999–2013	Sweden	Europe and Central Asia	HIC	III-3	Suicide where body mass index was known	18–70+	X			X	
Yang et al., 2018 [55]	2008–2015	South Korea	East Asia and Pacific	HIC	III-3	Bath-related deaths	18–91	X		X		
Youn et al., 2009 [56]	1998–2007	South Korea	East Asia and Pacific	HIC	III-3	OHCA due to drowning patents admitted to St Mary’s Hospital	3–87	X		X	X	

Abbreviations: HIC—high income country; LMIC—lower middle income country; LIC—low income country; OHCA—Out of Hospital Cardiac Arrest; UMIIC—upper middle income country; SUDEP—Sudden Unexpected Death in Epilepsy; UK—United Kingdom; Study evidence level: III-3 (comparative studies with concurrent controls and allocation not randomised (cohort studies), case control studies, or interrupted time series with a control group; Level IV (case studies with either post-test or pre-test/post-test outcomes).
